# ASHE careers—Dr. David J. Weber

**DOI:** 10.1017/ash.2025.10046

**Published:** 2025-08-07

**Authors:** David J. Weber

**Affiliations:** Division of Infectious Diseases, School of Medicine, University of North Carolina, Chapel Hill, USA

Dr. Weber currently serves as the Charles Addison and Elizabeth Ann Sanders Distinguished Professor of Medicine and Pediatrics, Professor of Epidemiology, Associate Chief Medical Officer, UNC Medical Center, and Medical Director, Department of Infection Prevention, UNC Medical Center, where he has led significant advancements in the science and prevention of healthcare-associated infections (HAIs). With decades of experience in research, education, and clinical practice, Dr. Weber has been a steadfast advocate for evidence-based solutions to combat emerging infectious diseases and antimicrobial resistance.

**You are well known among our society members in the global infection prevention and stewardship communities. Can you tell us briefly more about your background, and how you got your start in medicine, public health, and academics?**

I’ve always enjoyed science, and I was fortunate enough to be able to work in academic research labs every summer starting my sophomore year in high school and continuing all the way through college. Probably the two most interesting were the summer I spent at Scripps Institute of Oceanography after my sophomore year in high school, aiding a research team which was studying whether snakes can hear airborne sounds, and it was my job, since we had to use the same type of snake each time, to defang the rattlesnakes before each experiment. I should say the best parts of the summer was they taught me how to scuba dive at Scripps, and I got to dive once with Philippe Cousteau, who did all the photography for the National Geographic Specials, and I also got a chance to have dinner with the graduate student’s father, who I was working with, who was a Nobel Prize winner in 1967. So that was certainly exciting. Another interesting summer was spent at the Woodrow Wilson School at Princeton, aiding in research, and how children develop ideas about presidential leadership and politics. So, I’ve always enjoyed research. I always knew I wanted to teach in a at a university level, but I decided that I really didn’t want to spend my time just by myself in a laboratory. I enjoyed working with people too much, and I really wanted to improve public health. So ultimately I decided to become an infectious disease epidemiologist with the goal of splitting my time between a school of medicine and a school of public health, and I’ve certainly been fortunate enough over the last 41 years to be at the University of North Carolina, where the Medical school and the Gillings School of Global Public Health are right across the street from each other, and where I can split my time between teaching at the School of Public Health, and obviously doing infectious diseases and infection prevention at the School of Medicine

**What does mentorship and professional society membership mean to you?**

I’ve always been fortunate in having excellent mentors in infectious diseases including Drs. Mort Schwartz and Bob Moellering and excellent mentors in infection prevention that included Drs. Dennis Maki, Richard Wenzel, and Henry Masur in infection prevention. In fact, Dr. Masur convinced me while I was in my ID fellowship, as did Dennis Maki to go ahead and take my boards in critical care medicine, and once I got here at UNC, I did both adult and pediatric ID as well as adult critical care medicine. For many years, however, teaching and mentoring have been favorite activities. I’ve taught at the Gilling School of Global Public Health now for greater than 40 years and trained roughly 60 persons with either an MPH or a PhD. SHEA has allowed me to learn from my colleagues, and just as importantly, to mentor junior colleagues, both trainees and people earlier in their career. And that’s, as I’ve said, is my favorite activity. Again, mentoring impacts and improves my activities in public health, which I’ve always thoroughly enjoyed. I have particularly enjoyed the ability to be a member of greater than 100 SHEA Town Halls during the COVID-19 pandemic learning from my colleagues on the Town Halls, as well as answering questions from many of our colleagues around the country.

**You’re remarkably well published and knowledgeable on these matters. In an ideal world, how would hospital-based infection, prevention and antimicrobial resistance prevention strategies inform national policies and ongoing funding for innovations?**

I should first start off saying that working within a university and academic medical center has allowed me to teach trainees at many levels. Teaching has a multiplier effect. If the roughly 60 people I’ve trained have trained, each train 60 people, then we have thousands of new people, working to improve infection prevention, and public health in general. So that really to me what drove me to academics is that multiplier effect by training the next generation of infection preventionists, hospital epidemiologists, and infectious disease clinicians and researchers. Over the years, I’ve also been fortunate to train nurses and pharmacists through lectures, seminars, and workshops, as well as providing direct patient care, so I have thoroughly enjoyed my time in the Medical Center. It’s helped me grow as an individual. It’s always been fun. I also enjoy writing and publishing and I think that’s an important part of being in an academic center in terms of designing new research to improve infection prevention. In addition, most importantly, recently including implementation recommendations into all the guidelines developed by SHEA.I particularly enjoy having trainees serve as first author and allowing them to become part of the research community. More broadly, I value having served on several SHEA committees including Guidelines Committee, Awards Committee and Public Policy Committee. I’ve learned a great deal on those committees, and of course, that that given me the opportunity to have reached much further than individually publishing a paper. I think some of the things that SHEA has done such as the Town Halls, and guidelines in particular, are very important to our membership and to improving public health in general. I’ve been fortunate enough over the years to have served at the SHEA liaison on the ACIP from 2014 to 2020; as vaccines and vaccinology are my second major research interest because vaccines along with safe drinking water are the 2 greatest public health achievements in the last 150 years. More. recently I’ve been fortunate enough to serve on CDC’s HICPAC as well as several of HICPAC’s working groups, especially the occupational health and healthcare personal working group. Again, this is a way of improving the health of the public and spreading knowledge. Finally, I’ve been on several WHO working groups I also enjoy working with other infection prevention and hospital epidemiology, societies around the world, such as APSIC in Asia and Clean Hospitals in Geneva, and have found these a valuable learning experience for myself, and an important way of improving public health worldwide

**In your role as SHEA President, what are your primary goals for advancing infection, prevention, antimicrobial stewardship, and preparedness for emerging infectious diseases?**

Well, those are obviously 3 large and important areas for SHEA. I think one of the goals I personally have, and SHEA in grow the membership of SHEA, by attracting younger members, especially people in training to join SHEA. I think in addition, we want SHEA to attract members who are infectious disease pharmacists, infection prevention nurses, and PhD epidemiologists. I think that hospital epidemiology and infection prevention need to continue to evolve. The new paradigm is incorporate implementation science, QI, informatics, molecular epidemiology, AI and machine learning into our policies, procedures, and guidelines. I think we need to support the members early in their careers by allowing them to serve on SHEA committees and as SHEA liaisons to other professional organizations. I find it really astounding that roughly, 10% of all SHEA members serve on one of our committees. Additionally, Town Halls, training opportunities, journal clubs, and our SHEA courses should serve our current members and aim at increasing the number of people in SHEA, and particularly getting younger people involved in SHEA. In addition, SHEA continues to develop memorandums of understanding with other professional organizations, such as Clean Hospitals and APSIC and via liaisons with multiple other professional organizations, such as APIC, IDSA, WHO, ESCMID and others. One of the good things I think that we’re seeing forthcoming are the development of multi-society guidelines as opposed to single society guidelines. Nothing is more confusing to practicing clinicians than to have slightly different guidelines from multiple organizations. For instance, our recently published Sterilization, Disinfection guideline was led by SHEA, but was also endorsed by several other professional organizations and written with liaisons from the CDC, FDA, Joint Commission, and others. So, I think this will be an important new guideline. And as with all our guidelines, I think, including sections on implementation, is very important to our membership. Finally, I think SHEA, as an organization, wants to continue supporting our excellent journals and their excellent editors, ICHE and the newer journal, ASHE. Importantly, I think ASHE is the first journal, solely devoted to antibiotic stewardship and hospital epidemiology, and I think it’s a wonderful addition to SHEA and to science in general.

**Your SHEA presidency coincides one of the most challenging eras for health care and public health, where Federal policies directly challenge our daily work, and public trust in science and vaccines is actively undermined. How has this shaped your goals as SHEA President?**

I think SHEA must strive to promote evidence-based science in its guidelines and all its publications, in its journals, and when dealing with political sensitive issues. We need to support evidence-based science, to improve public health, and to improve the safety of patients. And we need to fight science denialism. Specifically, SHEA as an organization will continue to support CDC activities, especially those related to improving patient safety and infection prevention. It’s important for us to have a voice in developing CDC policies through committees such as HICPAC. We need to have input into NHSN (the National Healthcare Safety Network) and to ACIP, particularly regarding vaccines that promote the safety of our healthcare personnel. Separate from that we obviously need to be vigorous proponents of NIH funding both to improve infection, prevention and control and to address the increasing problem of antimicrobial resistance. It’s also important that we work with our other partners and professional organizations, such as IDSA to reach out across the globe to other groups.,. We should continue to work with these outside organizations, including the World Health Organization

**Are there any future collaborators that you may have on the horizon?**

Yes, I think we would certainly like to have increasing collaborations with the World Health Organization, particularly important as the United States disengages the Federal Government from the WHO, and I think we’d increasingly like to move to working with partners both in Europe through ESCMID, but as well, partners in the Middle East, in Africa, in South America and in Asia, and particularly playing a role in helping lesser developed and middle income countries improve their infection, prevention and control as well as aiding them in dealing with growing antimicrobial resistance and I think SHEA is an organization has great expertise that could aid these countries and their professional societies in developing better science and in dealing with these important public health problems

**What can SHEA journals, such as ICHE and ASHE, do to advance the mission of SHEA, and the science of infection, prevention, and antimicrobial stewardship?**

I think ICHE and ASHE already advance the mission of SHEA by publishing of first rate science, and of course ICHE, and now ASHE have always been my favorite journals in which to publish. I’ve had the fortune of publishing more than 220 papers in ICHE and ASHE, and I will continue to be vigorous proponents of the excellent science published by both journals. I think our membership clearly enjoys the close working relationship between the Guidelines Committee of SHEA and the SHEA journals, in addition, through the SHEA Compendium, we continue to provide new information on the core interests in infection prevention such as preventing central line associated infections, pneumonia, surgical site infections, and urinary tract infections, as well as now, developing totally new guidelines, such as diagnostic stewardship in the area of antimicrobial stewardship and prevention I would hope that SHEA and its journals will continue to be at the forefront of knowledge assessing health inequities. And of course, we need evidence-based science and public health to counter science denialism and vaccine hesitancy.

**Finally, what are some of the outside interests and hobbies that sustain you in challenging times?**

When I am traveling, I don’t really read scientific journals while I’m sitting on the plane or at the airport, rather I really enjoy reading fantasy and science fiction. I should mention that, of course, my favorite author in that area is David Weber. I do leave his book on my coffee table. I had nothing to do with writing it, of course, but I do find it very coincidental that the author, David Weber’s wife, is named Sharon, which is also my wife’s name. So many people, when they read that inscription to his wife that says to my beloved wife, Sharon, think I was the author, but I’m not. But I also enjoy reading other science fiction and fantasy novels as well. I also enjoy visiting my 3 grown daughters and including my 4 grandchildren with one just born in early April. In fact, if I knew how much more fun it was to be a grandfather than a father, I would have done it first, because it’s got all the advantages and none of the disadvantages of being a parent. Finally, my wife and I really enjoy traveling to foreign countries, particular places where we haven’t been. Recently we went to the Galapagos, had a wonderful time there, snorkeling and doing walking tours of the islands. One of the things I hope to get more involved with, and particularly once I retire, which is not imminent, is cooking. I’ve always enjoyed cooking and one of the things we’ve done while we travel is take local cooking classes. Usually, we start at the local market for an hour or 2, learn all about the foods at these locales, and then we pick the food with instruction. Then, we learn to cook wonderful foods, using new spices and new ingredients.

**Currently, you hear about the decreasing interest in infectious diseases, infection prevention, antimicrobial stewardship. What advice do you have for young doctors, or even medical students, or even trainees, who are considering a career in infectious diseases, or even young faculty members or young infectious disease specialists to keep them resilient and keep them engaged?**

Let me start off to tell you a little story about myself. When I was an ID fellow at Massachusetts General Hospital, I decided, as I said, to become a public health epidemiologist, and as part of that I thought I needed to get a master’s in epidemiology. So, I went and interviewed with Dr. Brian MacMahon, a famous cancer epidemiologist who was Chair of the Epidemiology Department at Harvard’s School of Public Health. He said to me when I explained that I wanted to be an infectious disease epidemiologist, and this is a direct quote, “son, this is a bad idea. We know everything there is to know about infectious diseases, and it should only be read about in the history books.” And then he said, “there’s no place in academic public health for infectious diseases anymore.” There was a belief at this time by some that antibiotics would cure all the bacterial diseases and vaccines would prevent all the viral diseases, so there was no longer a need to study the epidemiology of infectious diseases. Obviously, I did not listen to this advice. So, my advice remains the same, infectious diseases is an exciting field for clinical care and research. It’s never a question of if we’ll see another pandemic it’s only a question of when. The body of knowledge for us in infectious diseases is about 1,500 human infectious diseases. In the past 100 years, we’ve substantially lowered the morbidity and mortality due to infectious diseases. Vaccines have been tremendously effective in reducing morbidity and mortality worldwide, but we’re constantly faced with new diseases. However, we still face challenges to continued reductions in lower the morbidity and mortality of infectious diseases. For example, at the present time we have the largest measles outbreak in decades. We’ve had measles deaths for the first time since 2015. This is largely from increasing vaccine hesitancy. We have diseases increasing in numbers and expanding geographically, such as Dengue and Oropouche in South America that will clearly impact the United States. We often have outbreaks of viral hemorrhagic fevers in Africa. We need to remember that we are only a plane ride away from any of these infectious diseases. So, I personally think infectious diseases remain incredibly interesting, important, and fun to study. And I value the fact that, unlike many other areas of medicine, we not only prolong life and make people healthier but usually cure our patient. Further, we can prevent infections for an increasing number of infectious diseases via vaccines and other public health interventions such as safe drinking water and mosquito nets. I would encourage younger people to study infectious diseases with a focus on infection prevention and antimicrobial stewardship because it is an area that will continue to grow and become increasingly important. Plus, providing patient care, teaching, and conducting research is both fun and highly rewarding. Our goal in infection prevention is to eliminate healthcare-associated infections. We’ve done a good job in reducing our number of healthcare associated infections, but we still have a long way to go to get to 0, and of course we must increasingly deal with antimicrobial resistant microbes. Thus, we need to encourage trainees to enter the important fields of infection prevention and control, and antimicrobial stewardship.

